# Dickkopf-1 negatively regulates the expression of osteoprotegerin, a key osteoclastogenesis inhibitor, by sequestering Lrp6 in primary and metastatic lytic bone lesions

**DOI:** 10.1097/MD.0000000000003767

**Published:** 2016-06-17

**Authors:** Jian-Hang Wang, Yuanjin Zhang, Hong-Yan Li, Yun-Yan Liu, Tao Sun

**Affiliations:** a Trauma Department of Orthopedics, Yantaishan Hospital, Yantai, Shandong, China; b Department of Orthopedics, Huangshi Central Hospital, Affiliated Hospital of Hubei Polytechnic University, Huangshi, Hubei, China.

**Keywords:** bone pain, bone resorption, negative regulator, osteoclast, osteolysis, Wnt signaling

## Abstract

Recently, an inverse role for Wnt signaling in the development of osteoclasts in the bone was demonstrated. In the present study, we examined whether there is a commonality in the mechanism of bone resorption and lysis that occur in a diverse set of bone metastatic lesions, as well as in primary bone lesions. Compared with control bone tissue and bone biopsies from patients with nonmetastatic primary tumors (i.e., breast carcinoma, lung adenocarcinoma, and prostate carcinoma), patients with bone metastatic lesions from the three aforementioned primary tumors, as well as osteolytic lesions obtained from the bone biopsies of patients with multiple myeloma, demonstrated an upregulated expression of the glycoprotein Dickkopf-1 at both the mRNA and protein levels. Additionally, by coimmunoprecipitation, Dickkopf-1 pulled-down low-density lipoprotein receptor-related protein 6 (Lrp6), which is a key downstream effector of the Wnt signaling pathway. The expression of Lrp6 was unaltered in the osteometastatic lesions. This negative regulation was associated with a lowered expression of osteoprotegerin in the osteometastatic lesions, an observation that was previously reported to promote osteoclastogenesis. These findings provide a common mechanism for the inverse relationship between the Wnt signaling pathway and the development of primary or metastatic bone lesions. Pharmacological modulation of the Wnt signaling pathway might benefit the clinical management of primary and metastatic bone lesions.

## 1 Introduction

The *Wnt* gene family is comprised of a number of secreted proteins that have a highly conserved glycosylation pattern. Wnt signaling is complex because of the involvement of various ligands, receptors, and signaling pathways.^[1]^ Wnt receptors are typically members of the frizzled family of membrane-associated proteins on target cells. The binding of Wnt to its receptor leads to the formation of a complex with one of the low-density lipoprotein receptor-related protein (Lrp) coreceptors, which are primarily Lrp5/6 and disheveled.^[2]^ Recent evidence suggests an inverse role for Wnt signaling in the development of osteoclasts in the bone.^[3]^ This role was observed in normal physiology as well as in pathophysiological settings.^[4–9]^

Dickkopf proteins are negative regulators of the Wnt signaling pathway that interact with the cell surface membrane component of Lrp5/6 to sequester the protein. The formation of this complex leads to the internalization, ubiquitination, and proteosomal degradation of Lrp5/6. The destruction of Lrp is what inhibits Wnt signaling in the cell.^[2]^ Recently, it was demonstrated that Dickkopf-1 is deregulated in a wide variety of tumors, including circulating tumors, solid tumors, soft tissue tumors, and hard lesions.^[10–12]^

Wnt signaling in osteoblasts suppresses osteoclast function, which likely occurs through the Wnt-mediated production of osteoprotegerin.^[13]^ Osteoprotegerin functions as a decoy receptor for a key mediator of osteoclastogenesis, which causes the suppression of bone resorption. Similarly, Wnt may act directly on the osteoclast, leading to the same effect of decreased osteoclastic activity and bone resorption.^[14]^

It is well known that metastatic bone lesions from primary tumors that occur elsewhere, including breast, prostate, or lung adenocarcinoma, are notorious for altering circulating plasma calcium levels.^[15,16]^ Additionally, these metastases cause cancer-associated pain, which is one of the most distressing features of cancer. In the present study, we examined whether there is a commonality in the mechanism of bone resorption and lysis that occur in a diverse set of bone metastatic lesions, as well as in primary bone lesions. For the latter, we chose multiple myeloma as a model. Using these samples and age-matched controls, we examined the expression of the key Wnt signaling pathway molecule glycoprotein Dickkopf-1.^[17,18]^ The aim of the present study was to examine whether the reported inverse relationship between Wnt signaling and osteoclastogenesis is a universal molecular feature of bone metastatic lesions. We used a wide variety of metastatic and nonmetastatic bone biopsies to obtain evidence to support our hypothesis.

## 2 Materials and methods

### 2.1 Human bone biopsies from patients with nonmetastatic tumors and metastatic tumors to the bone

Patient and/or family consent was obtained before the study. Explicit permission was obtained from the Institutional Review Board of Yantaishan Hospital, China, and all of the experiments were conducted according to Helsinki guidelines with fully informed patient consent. Both male and female patients were enrolled into the present study, and the age ranges were between 52 and 77 years of age. Age-matched samples were used for control studies. The bone biopsy samples of each group were pooled, and the studies were conducted in triplicates for each condition with a total of three patients in each group. Bone biopsies were obtained from the multiple myeloma patients and the patients with primary tumors of the breast, lung, or prostate for diagnosis of the metastatic lesions in the bone. The control biopsies were obtained from patients with nonmetastatic disease. The bone biopsies were performed in these patients because of complaints of chronic bone pain and radiology-negative evidence of any bone lesion. The biopsy samples were stored at –20°C until further analyses. Similar protocols were followed for all of the experimental procedures to obtain unbiased and rigorous evidence.

### 2.2 Antibodies and chemicals

All of the antibodies were obtained from Abcam, RND Biotech, and Santa Cruz Biotechnology, and the chemicals were from Aldrich.

### 2.3 RNA harvesting and reverse transcription

Total RNA was prepared from the lysates with the aid of the RNeasy Mini kit (Qiagen, Valencia, CA, USA). All of the reactions were performed on ice. cDNA was reverse transcribed using the QuantiTect Reverse Transcription kit (Qiagen). The experiments were done in nuclease-free conditions to ensure the integrity of the nucleic acids.

### 2.4 RT-PCR to detect the expression levels of mRNA

Real-time quantitative PCR was performed using the ABI 7000 SDS incorporating Taqman probe mixture (Applied Biosystems, Foster City, CA, USA). The probes/primers for the analyzing the genes related to Dickkopf expression were designed with Primer Express 2.0 software. The oligonucleotide probes were tagged with the reporter dye 6-FAM at its 5′ end and the quencher TAMRA dye at the 3′ terminal end. The internal control was the housekeeping gene, glyceraldehyde-3-phosphate dehydrogenase (GAPDH). The PCR reaction was performed with the routine 45 cycles. The expression levels of the genes in the different disease conditions were identified with reference to the changes in comparison to the absolute expression levels of mRNA in the control bone biopsies.

### 2.5 Western blotting

To detect protein expression, the protein lysates were subjected to sodium dodecyl sulfate (SDS)-electrophoresis using 12% polyacrylamide gel electrophoresis (PAGE) gels. Extensive pilot experiments were performed to determine the time and voltage of separation, and each blot was run several times to optimize the run conditions. After electrophoresis, the gels were transferred onto polyvinylidene fluoride (PVDF) membranes. The indicated primary antibodies (at 1 to 50–1000 dilutions, as determined by extensive pilot testing) were incubated with the membranes. The horseradish peroxidase (HRP) secondary antibodies were always used at a 10-fold dilution. The chemiluminescent signal was detected using the ECL System (GE HealthCare), and the signal was developed in a dark room using a Kodak x-ray.

### 2.6 Coimmunoprecipitation

Tissue lysates were prepared with 1% TX-100 buffer containing 150 mM NaCl, Tris-HCL at pH 8, and 1% TX-100 supplemented with 1 mM MgCl_2_ and CaCl_2_. The lysates were incubated with the anti-Dickkopf-1 antibody (1.5 μg/mL) or the anti-Lrp6 antibody (1.5 μg/mL) with Protein A/G PLUS agarose beads (Santa Cruz) in a cold room for 6 hours. The immunoprecipitates were repeatedly washed with TX-100 buffer. Finally, the pulled-down proteins were eluted by boiling for a few minutes in SDS sample buffer. The pulled-down proteins were electrophoresed by SDS-PAGE, and a Western blot was performed using anti-Dickkopf-1 and anti-Lrp6 antibodies. The specificity of primary antibodies was examined and the specificity of the coimmunoprecipitation was determined by omitting either the primary antibody or the IgG control, respectively.

## 3 Statistics

The data are expressed as the means ± SEM. Comparisons between multiple groups were performed by analyses of variance (ANOVA).

## 4 Results

### 4.1 Increased mRNA expression for Dickkopf-1 in the metastatic bone lesions

**Figure 1:**
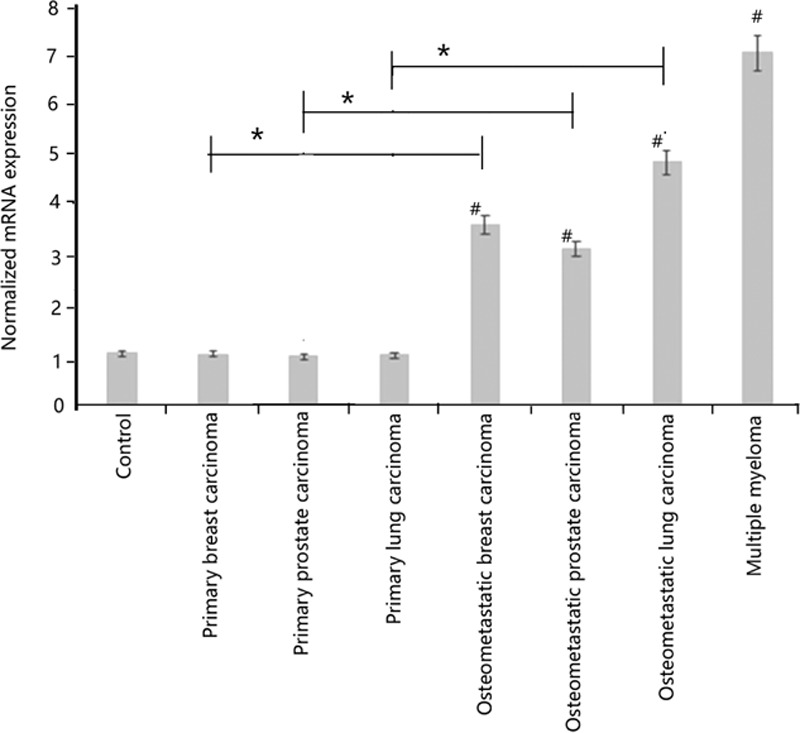
Histogram depicting alteration of the mRNA transcripts of Dickkopf-1 in the metastatic bone lesions and multiple myeloma. The normalized mRNA expression was estimated from the different transcripts from the standard curves. The primary tumors had no evidence of radiologic lesions but were biopsied because of persistent complaints of bone pain. The differences in the means between the groups were statistically significant when compared by analyses of variance. mRNA, messenger ribonucleic acid, #Metastatic bone lesion versus control (*P* <0.01); ∗metastatic bone lesion versus its corresponding primary tumor (*P* <0.01).

Compared with control bone tissues and bone biopsies from patients with nonmetastatic primary tumors (i.e., breast carcinoma, lung adenocarcinoma, and prostate carcinoma), patients with bone metastatic lesions from the 3 aforementioned primary tumors, as well as osteolytic lesions obtained from the bone biopsies of patients with multiple myeloma demonstrated an upregulated mRNA expression of the glycoprotein Dickkopf-1. The differences in the means between the groups were statistically significant when compared by analyses of variance (*P* <0.01) (Fig. 1).

### 4.2 Increased Dickkopf-1 protein expression in the metastatic bone lesions

**Figure 2:**
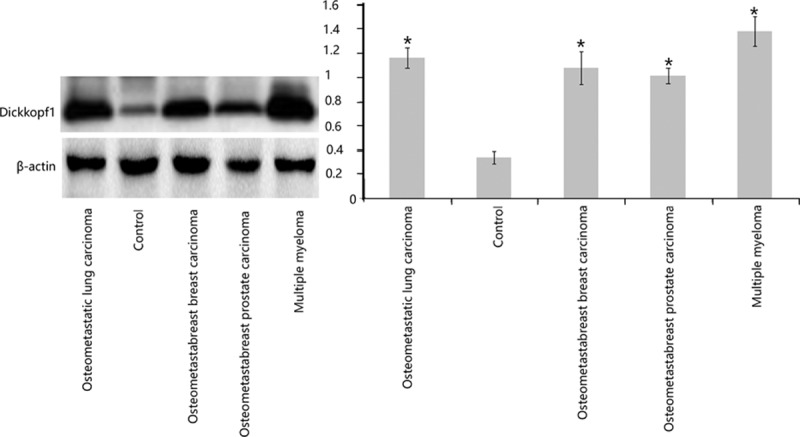
Western blot showing elevated levels of Dickkopf-1 in the osteometastatic lesions compared with the controls A representative blot is shown here. Triplicate samples were analyzed to obtain the quantitative intensities of the protein signals for Dickkopf-1 and β-actin (the loading control). These means between the groups were different and were statistically significant when examined by analyses of variance (∗*P* <0.01).

Compared with the control bone tissues, the bone metastatic lesions from the breast carcinoma, lung adenocarcinoma, and prostate carcinoma patients, as well as the osteolytic lesions obtained from the bone biopsies of patients with multiple myeloma, demonstrated an upregulated expression of the glycoprotein Dickkopf-1. Representative Western blots are shown in Fig. 2. Triplicate samples were analyzed for obtaining the quantitative intensities of the protein signals. These means between the groups were different and were statistically significant when examined by analyses of variance (*P* <0.01).

### 4.3 Coimmunoprecipitation shows an increased sequestration of Lrp6 by Dickkopf-1 in the metastatic bone lesions

**Figure 3:**
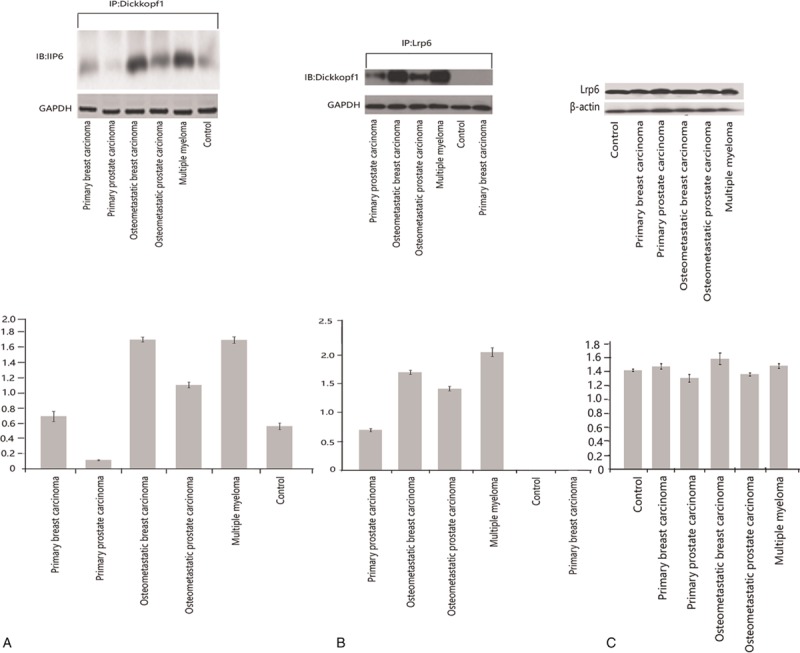
(A and B) Coimmunoprecipitation of the pulled-down Dickkopf-1–Lrp6 immunoblots with either antibody shows the binding. (C) Unaltered levels of Lrp6 in the osteometastatic lesions compared with the controls. All of the panels are representative Western blots. (A and B) In the far left and middle panel, the immunoprecipitated lysates were blotted for Dickkopfl-1 and Lrp6, respectively. GAPDH was used to demonstrate equal loading of the loading lanes. The histograms below each blot are the quantitative intensities of the protein signals. (C) The far right panel shows the expression of Lrp6 in the control, primary, and metastatic lysates. β-actin was used as a loading control. The histogram below represents the quantified intensities of the protein signals. All of the assays were performed in triplicate from pooled samples. GAPDH = glyceraldehyde-3-phosphate dehydrogenase.

Binding assays were performed to estimate the key signaling molecules for the Wnt signaling pathway, namely Lrp6. For this assay, the binding of Lrp6 with Dickkopf-1 was assayed by coimmunoprecipitation. In all of the metastatic bone lesions, an enhanced binding of Lrp6–Dickkopf-1 was observed when the proteins were immunoprecipitated with either the Lrp6 or the Dickkopf-1 antibody (Fig. 3A and B). Control experiments, in which the primary antibodies were omitted, did not result in the pull-down of the associated protein (data not shown). Minimal binding was seen in the primary tumors without metastasis (primary tumors of the breast/lung/prostate) and in the control bone biopsies. The expression of Lrp6 in the metastatic lesions was not different from the control tissues (Fig. 3C). This observation suggests that the increased sequestration of Lrp6 by the enhanced Dickkopf-1 expression probably results in the downregulation or inhibition of Wnt signaling.

### 4.4 Decreased protein expression of osteoprotegerin in the metastatic bone lesions

**Figure 4:**
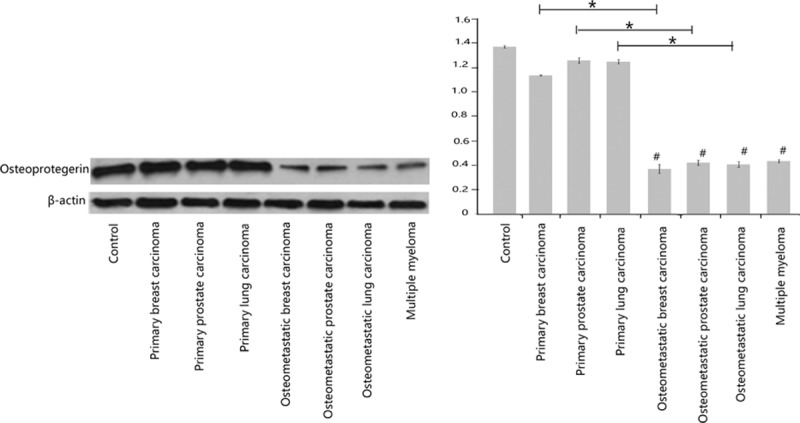
Western blots depicting diminished levels of osteoprotegerin in the osteometastatic lesions are shown. A representative blot is shown here. Triplicate samples were analyzed to obtain the quantitative intensities of the protein signals for osteoprotegerin and β-actin (the loading control). The differences in the means between the groups were statistically significant when compared by analyses of variance. #Metastatic bone lesion versus control (*P* <0.01); ∗metastatic bone lesion versus its corresponding primary tumor (*P* <0.01).

Compared with the control bone tissues and the bone biopsies obtained from patients with nonmetastatic primary tumors (i.e., breast carcinoma, lung adenocarcinoma, and prostate carcinoma), patients with bone metastatic lesions from the 3 aforementioned primary tumors, as well as osteolytic lesions obtained from the bone biopsies of patients with multiple myeloma, demonstrated diminished expression of osteoprotegerin, which is a negative regulator of osteoclastogenesis. Representative Western blots are shown in Fig. 4.

## 5 Discussion

Uncovering the role of the Wnt pathway in bone metastases offers a great opportunity for advancing and developing clinical therapeutics to achieve clinical progress in established diseases, such as metastatic bone lesions. The results of the present study provide strong evidence of a common basis for the genesis of osteolytic lesions in primary as well as in metastatic bone lesions. Namely, the upregulation of a key negative regulator of the Wnt signaling pathway is a common molecular denominator of these lesions. This glycoprotein, Dickkopf-1, is persistently elevated in osteometastatic bone lesions from breast or prostate primaries, but not in the primary tumor themselves per se. Additionally, Dickkopf-1 was also upregulated in the bone lesions of multiple myeloma patients. Recently, preliminary evidence suggests a role for Dickkopf in necrotic bone lesions.^[19,20]^

We further demonstrated that Dickkopf-1 strongly pulled-down Lrp6, which is a key downstream molecule that prevents the destruction of beta-catenin in the Wnt signaling pathway. Likely, this sequestration results in beta-catenin destruction in osteolytic bone lesions and suppression of the Wnt signaling pathway. These observations were coupled with our other observation that in the osteolytic bone lesions there was a consistent decrease in osteoprotegerin, which is a key negative regulator of osteoclastogenesis.

Combined, these observations show the interaction of key nodal points of the Wnt pathway in the generation of osteolytic lesions.^[21]^ Identification of these nodal points provides a molecular pharmacological basis of the regulated enhancement of the Wnt pathway or the inhibition of osteoprotegerin expression to control existing bone lesions to prevent their development.^[22]^ In metastatic lesions, these may provide additional therapeutic benefits, although they do not directly address the root of the primary tumors themselves. Similar is the case for multiple myeloma. However, the prevention of osteolysis may be of tremendous benefit, both from the viewpoint of preventing the massive suffering from bone pain and the clinical conundrum associated with elevated calcium from all of these lesions.^[23–25]^

In most tumor cells the Wnt pathway is active. However, Wnt antagonists specifically affect the bone, where they decrease bone formation to a basal level as opposed to blocking it entirely.^[5]^ For patients with multiple myeloma, it is established that Dickkopf-1 is expressed by the myeloma cells and suppresses osteoblast function. Presently, a human neutralizing IgG1 anti-Dickkopf monoclonal antibody, called BQ880, is being investigated to determine its effect on multiple myeloma-related bone disease.^[26]^ The utilization of fully humanized antibodies against Dickkopf-1 permits the assessment of how these antibodies might inhibit mediators associated with the osteoclast activation that is established by the tumor cells. By inhibiting the osteoclasts, this might indirectly decrease tumor growth by reducing the unending cycle that is associated with bone lysis and tumor growth. In addition, Dickkopf-1 inhibition might also lead to increased osteoblast differentiation and maturation, which could restore bone growth and possibly inhibit tumor growth.^[2]^ The results of our study contribute to the understanding of the role of the Wnt pathway in bone metastasis and provide an opportunity for further advancing the development of clinical therapeutics. The relatively early events that lead to the development of bone metastases are the key for finding the right target to inhibit further growth and the dissemination of bone disease. It seems that the Wnt pathway is leading the way with respect to this aspect of the disease, and our results should be used to gain a better understanding of the Wnt pathway in the formation of bone lesions.
